# Chronic hyponatremia in patients with proximal femoral fractures after low energy trauma: A retrospective study in a level-1 trauma center

**DOI:** 10.1016/j.bonr.2019.100234

**Published:** 2019-12-06

**Authors:** Daniel Bernd Hoffmann, Christian Popescu, Marina Komrakova, Lena Welte, Dominik Saul, Wolfgang Lehmann, Thelonius Hawellek, Frank Timo Beil, Mohammed Dakna, Stephan Sehmisch

**Affiliations:** a Department of Trauma-, Orthopaedic- and Plastic Surgery, University Medical Center Goettingen, Goettingen, Germany; b Department of Medical Statistics, University Medical Center Goettingen, Goettingen, Germany

**Keywords:** Proximal femur fracture, Hyponatremia, Low energy trauma, Geriatric trauma, Electrolyte disorder, Hip arthroplasty

## Abstract

**Introduction:**

We evaluated the prevalence and influence of chronic hyponatremia in patients with low energy trauma. We also investigated the influence of medication and diseases on hyponatremia.

**Material and methods:**

This retrospective study included 314 cases of proximal femoral fracture due to low energy trauma. Patients were treated in the University Medical Center Goettingen within 3 years. Hyponatremia was defined as serum sodium <135 mmol/L at admission.

**Results:**

Overall, 15.6% of patients in the low energy trauma group had hyponatremia. Among patients older than 80 years, women showed distinctly higher rates of hyponatremia (female: 16.4%; male: 5.9%). In contrast only 4.7% of patients who underwent elective hip arthroplasty showed hyponatremia. Patients on sartanes and aldosterone antagonists showed significantly higher rates of hyponatremia. Alcoholism was significantly associated with hyponatremia.

**Conclusions:**

We confirmed a high prevalence of chronic hyponatremia in patients with fractures due to low energy trauma. Our data underscore chronic hyponatremia as a contributing factor to hip fractures. Women older than 80 have a higher risk of developing hyponatremia. Sartanes, aldosterone antagonists, and alcohol disease are associated with hyponatremia. Treating hyponatremia may decrease the risk of fracture after low energy trauma. Therefore, physicians of different specialties should focus on treatment of chronic hyponatremia to reduce the fracture rate associated with low energy trauma.

## Highlights

• 15.6% of patients with prox. femur fracture after low energy trauma had hyponatremia.
• Hyponatremia rate in group of patients before hip arthroplasty was only 4.7%.
• Sartanes, aldosterone antagonists, and alcohol disease are associated with hyponatremia.
• Women older than 80 have a higher risk of developing hyponatremia.

## 1 Introduction

Osteoporosis remains a complex challenge for modern medicine, despite promising treatment options in recent years. Several studies predict an enormous increase in the incidence of osteoporosis and associated fractures in the next decades, because of demographic trends (Osterkamp, 2005; Duquet, 2014). Therefore, more studies about the basic principles of the development of osteoporosis and its treatment are needed.

The most prevalent type of osteoporosis, postmenopausal osteoporosis (Type I), is a result of estradiol deficiency in women after menopause (Pacifici, 1998). Besides postmenopausal osteoporosis there are several variants of osteoporosis, e.g., senile osteoporosis (Type II) as a result of ageing bones, idiopathic osteoporosis, or medication-induced osteoporosis (Riggs, 1979; Heshmati and Khosla, 1998; Mirza and Canalis, 2015). The trabecular bones are primarily affected by osteoporosis, e.g., the distal radius, proximal femur, or vertebrae. Besides immediate genetic, hormonal, or medication-induced (e.g., glucocorticoids) factors, there are several further influencing factors on bone disorders like alcohol, nicotine, or endocrine factors (Strom et al., 2011).

Hyponatremia is the most common electrolyte disorder in elderly people (Cumming et al., 2014). It is defined as serum sodium lower than 135 mmol/L (Spasovski et al., 2014). Chronic hyponatremia is associated with higher risks for falls, attention deficits, and unsteadiness (Renneboog et al., 2006). In a rat model, a significant reduction of bone mass, of approximately 30%, resulted from chronic hyponatremia (Verbalis et al., 2010). Trabecular and cortical bone properties decreased as a result of chronic hyponatremia (Verbalis et al., 2010). Furthermore, several clinical trials indicate a significant prevalence of hyponatremia in elderly patients with low energy traumata and an association between chronic hyponatremia and osteoporosis (Cumming et al., 2014; Verbalis et al., 2010; Tolouian et al., 2012; Gankam Kengne et al., 2008; Ayus and Arieff, 1999). In fact, Ayus et al. showed already in 1999 that orthopaedic injuries can be the initial presentation of chronic hyponatremia in post-menopausal women (Ayus and Arieff, 1999). Moreover a recent study of Ayus et al. from 2016 indicated that mild hyponatremia in the elderly is an independent risk factor for hip fractures (Ayus et al., 1999). Effects of hyponatremia are varying from asymptomatic to hyponatremic encephalopathy and death. Risk factors in hyponatremic patients are alcoholism, hypoxia, female gender and children (Ayus et al., 2016; Ayus et al., 1992; Arieff et al., 1992).

The aim of this study was to evaluate the prevalence and influence of chronic hyponatremia on patients with fractures related to osteoporosis. For this reason we investigated the serum sodium levels in patients with proximal femoral fractures after low energy trauma. Within this scope, we aimed to investigate the influence of medication and diseases on hyponatremia in these patients.

## 2 Materials and methods

### 2.1 General procedures

The present study is a retrospective study of all patients older than 18 years with a proximal femoral fracture due to low energy trauma, who were treated in the University Medical Center Goettingen between Jan 1, 2010 and Dec 12, 2012. The study was approved by the ethical committee of the University Medical Center Goettingen.

Low energy trauma was defined as falls from standing height or less than 1 m and fracture without trauma.

To compare these data we used two control groups. On the one hand we included patients with proximal femoral fractures due to high energy trauma, e.g., road accident, fall from a great height, or bicycle accident. On the other hand we included patients as second control group, who underwent a total hip arthroplasty. These patients received a hip replacement due to osteoarthritis or aseptic hip prosthesis loosening. Patients with fractures, cancer or septic prosthesis loosening were excluded in this group.

Hyponatremia was defined as serum sodium level lower than 135 mmol/L. The serum sodium level at admission was the essential determining factor for classification as hyponatremia.

We identified age, weight, body height, diseases, gender, and medication of the patients. We investigated if patients were taking selective serotonin reuptake inhibitors (SSRI), thiazides, loop diuretics, proton-pump inhibitors (PPI), angiotensin-converting enzyme inhibitors (ACE-Inhibitors), sartanes, and aldosterone antagonists.

### 2.2 Statistical analysis

Baseline variables were compared between the high and low energy trauma groups, and for the low energy trauma cohort, between normo- and hyponatremia respectively. For the binary variables, Fisher's exact test and the*χ*2test for categorical variables were used. For continuous variables, a Shapiro-Wilk Normality Test was conducted to test the normality of the data.

All the continuous data were normally distributed; therefore, a *t* − test was performed to assess differences between groups. Factors that showed an association (defined by a *p*-value <0.2 in either the Fisher or *t*-test) were further combined in a multivariate logistic model to assess their effect on the dependent variable hyponatremia, coded as a binary outcome (no hyponatremia = “-” and hyponatremia = “+”). A bootstrap cross-validation of the resulting model was conducted to assess its validity.

For the single tests, the significance level was set to α = 5% for all statistical tests. It should be noted that when testing multiple hypotheses at the same time, an inflation of the α error occurs and must be accounted for with proper correction. All analyses were performed with the statistical software R (version 3.4.0, www.r-project.org).

### 2.3 Data

The data set was divided in two parts. The first part contained 42 patients, referred to as those with high energy trauma (the controls). The second part contained 314 patients, referred to as those with low energy trauma.

## 3 Results

### 3.1 Subjects

Within the study period, we found 360 cases of proximal femoral fractures due to low energy trauma. In 36 cases, the required data were not available. These patients were excluded. Furthermore, 10 patients were excluded because of cancer-induced bone metastases. The study included 314 cases from 306 patients. Eight patients had two independent proximal femur fractures in the time period.

As first control group, we used 42 patients with proximal femoral fractures due to high energy trauma. As second control group we used 126 patients before hip arthroplasty due to osteoarthritis or aseptic hip prosthesis loosening.

### 3.2 Comparison between patients with low energy trauma, high energy trauma and hip arthroplasty

Patients with high energy trauma were significant younger (52 years±15.2) than patients with low energy trauma (77.5 years ±11.6) (data not shown). 35.7% of patients were female in the high energy trauma-group, compared with 62.1% in the low energy trauma group. Overall, serum sodium levels were only slightly decreased in the low energy trauma group compared with the high energy trauma group (low energy trauma group: 138.1 mmol/L ± 4.7; high energy trauma group:140.8 mmol/L ± 2.7). In the high energy trauma group, no patient showed hyponatremia at admission. There were no significant differences in weight or body mass index between the two groups (data not shown).

Patients who underwent hip arthroplasty had an average age of 67.4 years (±11.4). 39% of patients were male, 61% female in this group. Average serum sodium levels were between both trauma groups (139.3 mmol/L ± 2.5).

### 3.3 Hyponatremia in low energy trauma group and hip arthroplasty group

**Fig. 1:**
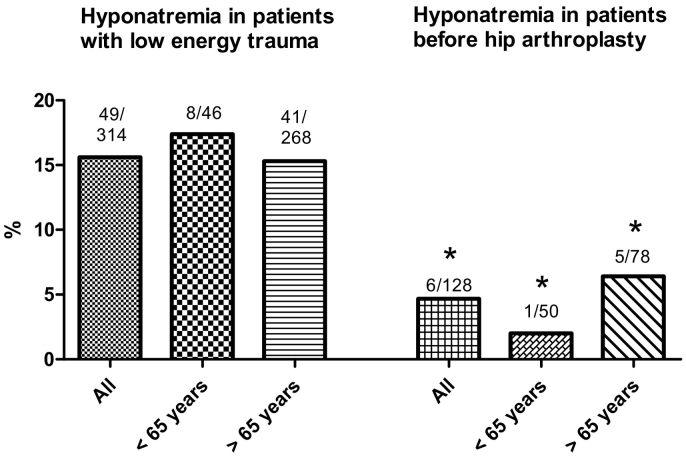
Hyponatremia in patients with low energy trauma and before hip arthroplasty. Number of patients with hyponatremia and total number in the cohort are written above the bars. Overall 15.6% of all patients in low energy trauma group showed hyponatremia at admission. In the group of patients <65 years, 17.4% (8 of 46 patients) showed hyponatremia at admission. Patients >65 years showed hyponatremia in 15.3% (41 of 268 patients). Hyponatremia rate in group of patients before hip arthroplasty was only 4.7%. In the age cohort <65 years 2% of patients had hyponatremia (1 of 50 patients). In the group of patients >65 years 6.4% showed hyponatremia (5 of 78 patients). * = *p* < 0.05 vs. low energy trauma group

Overall, 15.6% (49 of 314) of all patients in the low energy trauma group showed hyponatremia at admission (Fig. 1). The average sodium serum level in this group was 129.9 mmol/L ± 4.1. 63.2% of these patients were female, and the mean age 77 years. Mean age and gender distribution did not differ significantly from patients in the group without hyponatremia (77.6 years, 61.9% female). There was no significant difference in body mass index between patients with or without hyponatremia (hyponatremia: 24.6 ± 3.9; no hyponatremia: 24.8 ± 4).

We separated patients with low energy trauma into two different age cohorts, younger and older than 65 years (Fig. 1). In the group of patients <65 years, 17.4% (8 of 46 patients) showed hyponatremia at admission. Patients >65 years showed hyponatremia in 15.3% (41 of 268 patients) of cases. The differences between both age cohorts were not statistically significant.

In the hip arthroplasty group six patients showed hyponatremia at admission (4.7%). In the age cohort <65 years 2% of patients had hyponatremia (1 of 50 patients). In the group of patients >65 years 6.4% showed hyponatremia (5 of 78 patients). The differences between arthroplasty groups and low energy trauma groups were statistically significant (Fig. 1).

### 3.4 Gender-related hyponatremia in low energy trauma group

Overall, in the low energy trauma group, the gender distribution of the hyponatremia group (female 61.9%, 164 of 265 patients) and non-hyponatremia group (female 63.2%, 31 of 49 patients) was almost equal.

In the age cohort <65 years males showed a non-significant higher rate of hyponatremia (female: 6.7%, male: 22.6%) in the low energy trauma group. In the group of patients >65 years female patients showed a higher rate of hyponatremia (female: 16.6%, male: 12.5%), but also with no statistical difference.

**Fig. 2:**
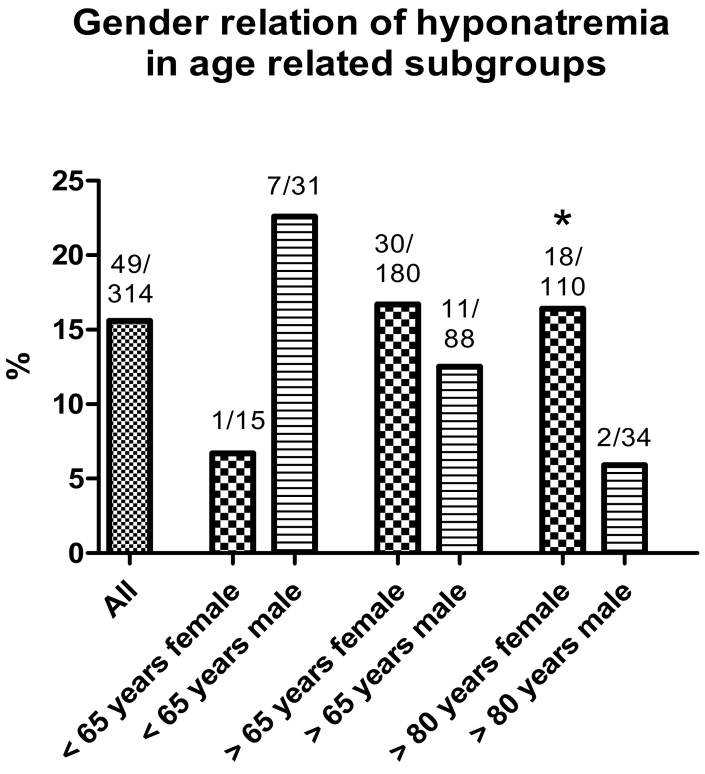
Gender distribution of hyponatremia in age related subgroups in low energy trauma group. Number of males or females with hyponatremia and total number in the cohort are written above the bars. Rate of hyponatremia between female and male patients did not differ significantly in groups older and younger 65 years. In a subgroup analysis with patients over 80 years, female patients showed significantly higher rates of hyponatremia than males (female: 16.4%, male: 5.9%). * = p < 0.05 vs. male

However, in a subgroup analysis with patients over 80 years in the low energy trauma group, female patients showed significantly higher rates of hyponatremia than males (female: 16.4%, male: 5.9%) (Fig. 2).

### 3.5 Influence of medication on hyponatremia in low energy trauma group

#### 3.5.1 Hyponatremia related to medication

Patients using sartanes medication showed significantly higher rates of hyponatremia than patients without sartanes medication (sartanes: 28.2% hyponatremia; no sartanes: 13.8% hyponatremia; odds ratio (OR): 3.2). For patients using aldosterone antagonists, higher rates of hyponatremia were also shown (aldosterone antagonist medication: 25% hyponatremia; no aldosterone antagonist medication: 14.8% hyponatremia; OR: 2.9). Patients using SSRI, thiazides, and PPI medication showed only insignificantly higher rates of hyponatremia. Loop diuretics and ACE-inhibitors were only insignificantly associated with lower rates of hyponatremia (Table 1).

**Table 1:** Hyponatremia related to medication in low energy trauma group.

	No hyponatremia(%)	Hyponatremia(%)	OR	
SSRI-	85.5	14.5		
SSRI+	76.3	23.7		
AldosteronAntagonist-	85.2	14.8		
AldosteronAntagonist+	75.0⁎	25.0⁎	2.9	
ThiazideDiuretics-	85.1	14.9		
ThiazideDiuretics+	81.9	18.1		
Loop Diuretic-	82.3	17.7		
Loop Diuretic+	88.3	11.7		
Sartanes-	86.2	13.8		
Sartanes+	71.8⁎	28.2⁎	3.2	
ACE inhibitor-	82.7	17.3		
ACE inhibitor+	87.0	13.0		
PPI-	86.40	13.60		
PPI+	81.20	18.80		


Hyponatremia related to medication. Patients using sartanes and aldosterone antagonist medication showed increased rates of hyponatremia. Patients with SSRI, thiazides, and PPI medication showed only insignificantly higher rates of hyponatremia. Loop diuretics and ACE-inhibitors were only insignificantly associated with lower rates of hyponatremia.

⁎ = p < 0.05 vs. without medication. The *p*-values and the OR are obtained from the multiple regression model described in the text.

#### 3.5.2 Medication related to hyponatremia

Conversely, patients with hyponatremia showed significantly higher rates of use of sartanes than patients with no hyponatremia (hyponatremia: 22.4%; no hyponatremia: 10.6%) (Table 2). In patients using sartane medication, the rate of combined use of additional thiazide diuretics was similar in both groups (hyponatremia: 27.3%; no hyponatremia: 28.6%). There was no significant difference in use of the other drugs between the two groups (Table 2).

**Table 2:** Medication related to hyponatremia in low energy trauma group.

	Hyponatremia	No hyponatremia
SSRI	18.4%	10.9%
AldosteronAntagonist	12.2%	6.8%
ThiazideDiuretics	26.5%	22.3%
Loop Diuretic	26.5%	37.0%
Sartanes	22.4%⁎	10.6%
ACE inhibitor	32.6%	40.4%
PPI	44.9%	35.8%

Medication related to hyponatremia. Patients with hyponatremia showed significantly higher rates of use of sartanes medication. For use of the other drugs we did not find significant differences. * = p < 0.05 vs. Normonatremia.

⁎ = p < 0.05 vs. No hyponatremia

### 3.6 Influence of diseases on hyponatremia in low energy trauma group

Alcoholism was significantly associated with hyponatremia. 50% of patients with alcohol disease showed hyponatremia (OR: 8.5) (Table 3). Patients with renal failure (renal failure: 7.1% hyponatremia; no renal failure: 17.4% hyponatremia, OR: 0.3) or Type 2 diabetes mellitus (Type 2 DM) (Type 2 DM: 8.1% hyponatremia; no Type 2 DM: 17.9% hyponatremia; OR:2.5) showed significantly lower rates of hyponatremia than patients without these diseases (Table 3). A history of heart failure and hypothyroidism showed no significant association with hyponatremia.

**Table 3:** Influence of diseases on hyponatremia in low energy trauma group.

	No hyponatremia (%)	Hyponatremia (%)	OR
AlcoholAnamnesis -	85.8	14.2	
AlcoholAnamnesis +	50.0	50⁎	8.5
Hypothyreoidism -	84.1	15.9	
Hypothyreoidism +	88.0	12.0	
Heart failure -	84.2	15.8	
Heart failure +	85.7	14.3	
DM -	82.1	17.9	
DM +	91.9	8.1⁎	0.4
Renal failure -	82.6	17.4	
Renal failure +	92.9	7.1⁎	0.3

Alcoholism was significantly associated with hyponatremia. 50% of patients with alcohol disease showed hyponatremia. Patients with renal failure and diabetes mellitus showed significantly lower rates of hyponatremia than patients without these diseases. Heart failure and hypothyroidism showed no significant association with hyponatremia. * = p < 0.05 vs. without disease.

⁎ = p < .05 vs. without disease.

## 4 Discussion

Our data confirm findings of previous studies that hyponatremia is highly prevalent in patients with fractures due to low energy trauma (Cumming et al., 2014; Gankam Kengne et al., 2008; Sandhu et al., 2009; Kwak et al., 2015; Nigwekar et al., 2019). Overall, we found hyponatremia in 15.6% of patients after low energy trauma compared with 2–4% in the general population, and no case of hyponatremia in the control group with high energy trauma (Mohan et al., 2013; Mohan et al., 2013). The shown rate of hyponatremia in the hip arthroplasty group (4.7%) is comparable with hyponatremia in the general population. There were no significant differences in weight or body mass index between the low energy and high energy trauma groups as well as hip arthroplasty group.

In previous studies, prevalence of hyponatremia in patients with fractures at admission was indicated between 9% and 13.6% (Cumming et al., 2014; Gankam Kengne et al., 2008; Kwak et al., 2015; Kwak et al., 2015; Aicale et al., 2017). Most of these studies included only patients with a minimum age of at least 60 years. Adjusted to a minimum age of 60 years, the prevalence of hyponatremia in our study was 17.1%. It is important to note the point of time of sodium measurement. In the present and aforementioned studies, sodium levels at admission were the essential determining factor for classification as hyponatremia or no hyponatremia. In some studies, sodium levels were analyzed during the entire stay in hospital. This included post-surgical hyponatremia of short duration. In the present study we focused on the relationship between low energy fractures and chronic hyponatremia at admission. However, the present study is limited by the fact, that we are uncertain about the actual period of preexisting hyponatremia. In this study we declared hyponatremia at admission as chronic hyponatremia. Nevertheless there are also previous studies indicating that chronic hyponatremia is a major determinant of the risk of hip fracture (Ayus et al., 2016; Nigwekar et al., 2019).

In the control group with high energy trauma we did not observe any hyponatremia at admission. The relatively limited number of patients could be a limiting factor for general conclusions about this control group. However, rate of hyponatremia in arthroplasty group is comparable with hyponatremia in general population (Mohan et al., 2013; Kinsella et al., 2010).

In the present study the rate of hyponatremia between patients younger and older than 65 years in the group with fractures due to low energy trauma was almost equal. From the authors point of view this fact suggests also chronic hyponatremia as important independent risk factor for hip fractures.

Previous studies are inconsistent regarding the relationship between gender and cases of hyponatremia. Overall, in our study the gender ratios between hyponatremia and no hyponatremia groups were equal. Even when groups are divided into younger and older than 65 years, the differences between male and female patients were not significant. This confirms the results of a study by Hawkins et al. in patients with no fractures (Hawkins, 2003). However, in a sub-analysis with patients over 80 years, there are significant differences in the prevalence of hyponatremia between males and females. According to our results, there is a significantly increased risk for female patients having hyponatremia from the age of 80 and older compared with male patients. This is consistent with a study by Mohan et al. on patients with no fracture (Mohan et al., 2013).

The influence of medication on sodium levels was investigated in several previous studies (Cumming et al., 2014; Gankam Kengne et al., 2008; Sandhu et al., 2009; Correia et al., 2014; Peyro Saint Paul et al., 2014; Izzedine et al., 2002; Wilkinson et al., 1999). In most of the studies, the use of drugs in patients with hyponatremia was investigated. In the present study we also investigated the rate of hyponatremia for patients using different drugs. Patients who took aldosterone antagonists and sartanes showed significant higher rates of hyponatremia than patients who did not take these drugs. For thiazide, PPI, SSRI, loop diuretics, and ACE inhibitors we could not confirm significantly higher rates of hyponatremia. Conversely, patients with hyponatremia showed significantly higher rates of use of sartanes than other drugs. In summary, according to our results there is strong evidence for the influence of sartanes on hyponatremia. This influence of sartanes was statistically independent of combined use of thiazide diuretics. Our study confirms also aldosterone-antagonist-related hyponatremia and gives more evidence according to previous studies (Biswas and Davies, 2007; Arampatzis et al., 2013; Handler, 2008).

In contrast to our findings, there is good evidence for SSRI- and thiazide-related hyponatremia in several previous studies (Fabian et al., 2004; Jacob and Spinler, 2006; Mann, 2008; Clayton et al., 2006; Jung et al., 2011). In our study, we found higher rates of hyponatremia for both drugs, but with no statistical significance. From the authors' point of view this might be an issue of low statistical power and it might be that the differences would be significant if the groups had more patients.

In the present study, we could not confirm a significant influence of loop diuretics on chronic hyponatremia. Hyponatremia is possible but not that common due to loop diuretics (Sahay and Sahay, 2014). On the other hand there are also studies which show an influence on chronic hyponatremia from loop diuretics (Arampatzis et al., 2013).

In the present study, patients with diabetes mellitus or chronic kidney diseases with proximal femur fractures after low energy trauma showed lower rates of chronic hyponatremia than patients without these diseases. These findings are surprising, because in general, diabetes mellitus and chronic kidney disease are associated with hyponatremia (Liamis et al., 2014; Kovesdy, 2012). From the authors' point of view there are two reasons for these results. On the one hand, in the present retrospective study, we may have included patients with unknown and untreated diabetes mellitus or chronic kidney disease. This would cause a mismatch in classification of the patients. On the other hand, patients with known diabetes mellitus or chronic kidney diseases are treated according to the current guidelines, which aim to prevent symptoms like hyponatremia. Furthermore, hyponatremia is usually typical for acute decompensation of these diseases and is not a chronic symptom (Liamis et al., 2014).

According to our data, chronic heart failure had no influence on chronic hyponatremia in patients with proximal femur fracture after low energy trauma, although hyponatremia is often present in patients with heart failure (Filippatos and Elisaf, 2013).

Consistent with previous studies we could confirm alcohol disease as a significant risk factor for hyponatremia (Liamis et al., 2000). However, the small number of patients with alcohol disease in the present study is a limiting factor for conclusions.

## 5 Conclusions

In summary, we can confirm a high prevalence of chronic hyponatremia in patients with fractures after low energy trauma. Our data underscore chronic hyponatremia as a contributing factor to hip fractures. Female patients aged 80 years and older have a higher risk of developing hyponatremia. Sartanes, aldosterone antagonists, and alcohol disease are associated with hyponatremia. Chronic hyponatremia can increase the risk of low energy trauma fractures in two ways. On the one hand, by higher risks for falls, attention deficits, and unsteadiness. On the other hand, by reduction of bone mass due to decreased bone formation. Therefore, physicians of different specialties should be aware of this situation and should focus on treatment of chronic hyponatremia to reduce the low energy fracture rate.

## Declaration of competing interest

None.
